# Metagenomic data of bacterial 16S rRNA in the cemetery soil samples in Surakarta City, Indonesia

**DOI:** 10.1016/j.dib.2023.109963

**Published:** 2023-12-14

**Authors:** Triastuti Rahayu, Erma Musbita Tyastuti, Ambarwati Ambarwati, Lina Agustina, Noor Alis Setiyadi, Nazia Jamil, Yasir Sidiq

**Affiliations:** a Department of Biology Education, Faculty of Teacher Training and Education, Universitas Muhammadiyah Surakarta, Surakarta 57162, Indonesia; b Department of Public Health, Faculty of Health Science, Universitas Muhammadiyah Surakarta, Surakarta 57162, Indonesia; c Institute of Microbiology and Molecular Genetics, University of the Punjab, Lahore 54590, Pakistan

**Keywords:** Bacteria, Cemetery soil, Genomic, Sequence data

## Abstract

Cemetery soils most likely contain degradative bacteria which possibly have beneficial potencies. However, the bacterial exploration in these potencies is still limitedly conducted in Indonesia. The raw sequence data of total bacteria in the cemetery soils through metagenomic analysis have been revealed. The data were obtained by collecting soil samples from six spots of two major Cemetery areas, which were Pracimaloyo (P) and Bonoloyo (B), in Surakarta City, Central Java, Indonesia. The six sample spots consisted of two samples from P area with respectively 20 cm and 140 cm depths and four samples of each two samples from B area with 20 and 40 cm depths. The total DNA was subsequently extracted from the collected soils using ZymoBIOMICS DNA Miniprep Kit. The total DNA then was amplified using a couple of 16S rRNA primers through Illumina HiSeq 2500 PE250 (Novogen, Korea) environment system. The raw sequence data has been submitted to the National Center for Biotechnology Information (NCBI) with project ID PRJNA997385. The archived sequence can be accessed in the NCBI website with the following URLs https://www.ncbi.nlm.nih.gov/sra/PRJNA997385. A brief analysis of the sequence data showed that the most common phyla in 20 cm-depths were Proteobacteria (29.5%), Actinobacteria (21.6%), and Firmicutes (19.2%), while Actinobacteria were the most found in 140 cm-depths with 34.2% followed by Proteobacteria (21.9%) and Firmicutes (16.6%). This data would be the first report of total bacterial sequence from cemetery soils in Indonesia.

Specifications Table

**Table:** 

Subject	Environmental Genomics and Metagenomics
Specific subject area	16S rRNA metagenomic sequence of bacteria in the cemetery soil
Data format	Raw DNA sequence
Type of data	16S rRNA read sequences, table, figure
Data collection	The soil samples were collected from two major Cemetery grounds in Surakarta City, Indonesia, namely Pracimaloyo (P) and Bonoloyo (B). The collected soils were picked up from 20 cm depths (Group A) and 140 cm depths (Group B). Two soil samples were collected from P area and four samples were from B areas, for a total of six samples. DNA of the soil samples were extracted using ZymoBIOMICS DNA Miniprep Kit and were subjected for next generation sequencing using 16S rRNA primers.
Data source location	Pracimaloyo and Bonoloyo cemetery ground City: Surakarta, Central Java Country: Indonesia Coordinates for collected samples/data: 1. Pracimaloyo Cemetery ground: 7°33′49"S 110°46′30"E 2. Bonoloyo Cemetery ground: 7°32′16"S 110°49′32"E Altitudes:Point 1 (P 12) = 111.9 m aslPoint 2 (B 18) = 131 m aslPoint 3 (B 23) = 138.2 m asl
Data accessibility	Raw sequencing data were archived in the NCBI and can be accessed within this link https://www.ncbi.nlm.nih.gov/sra/PRJNA997385. Repository name: SRA-NCBI Data identification number: PRJNA997385 Direct URL to data: https://www.ncbi.nlm.nih.gov/sra/PRJNA997385

## 1 Value of the Data

• Data in this paper revealed the lack information of bacterial diversity in Cemetery ground in Indonesia.
• The metagenomic data serves total bacteria in the soil so that gives additional information to isolation method.
• The data suggests the presence of bacteria with decomposition capabilities suitable for diverse biotechnological applications.

## 2 Data Description

**Fig. 1:**
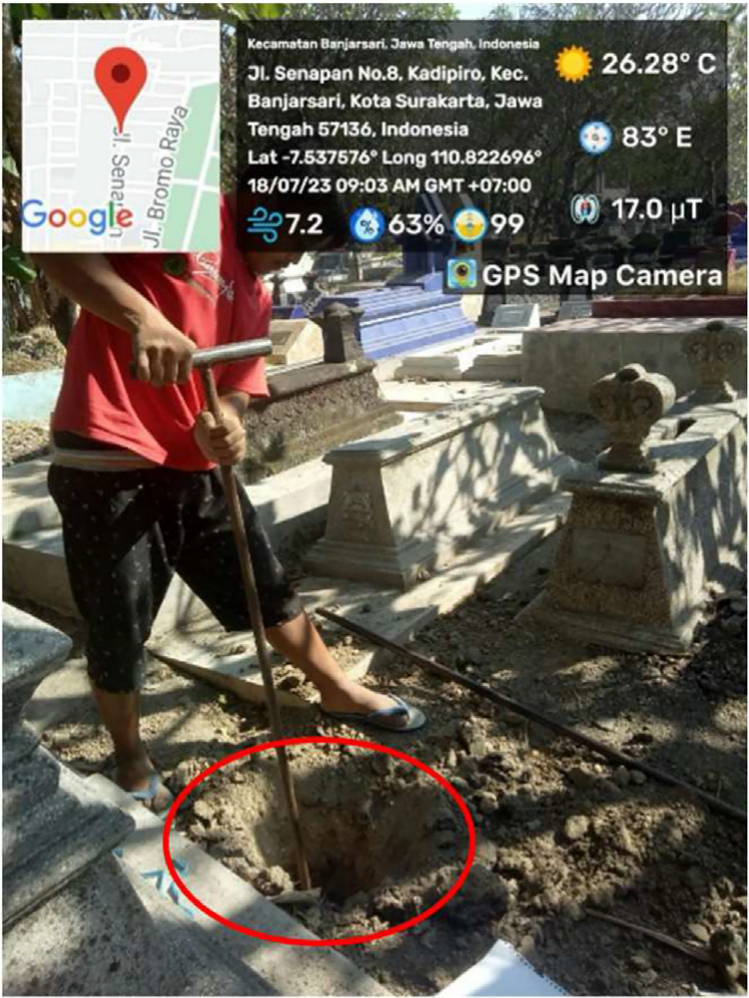
The representative photograph of sampling point (red circle).

**Fig. 2:**
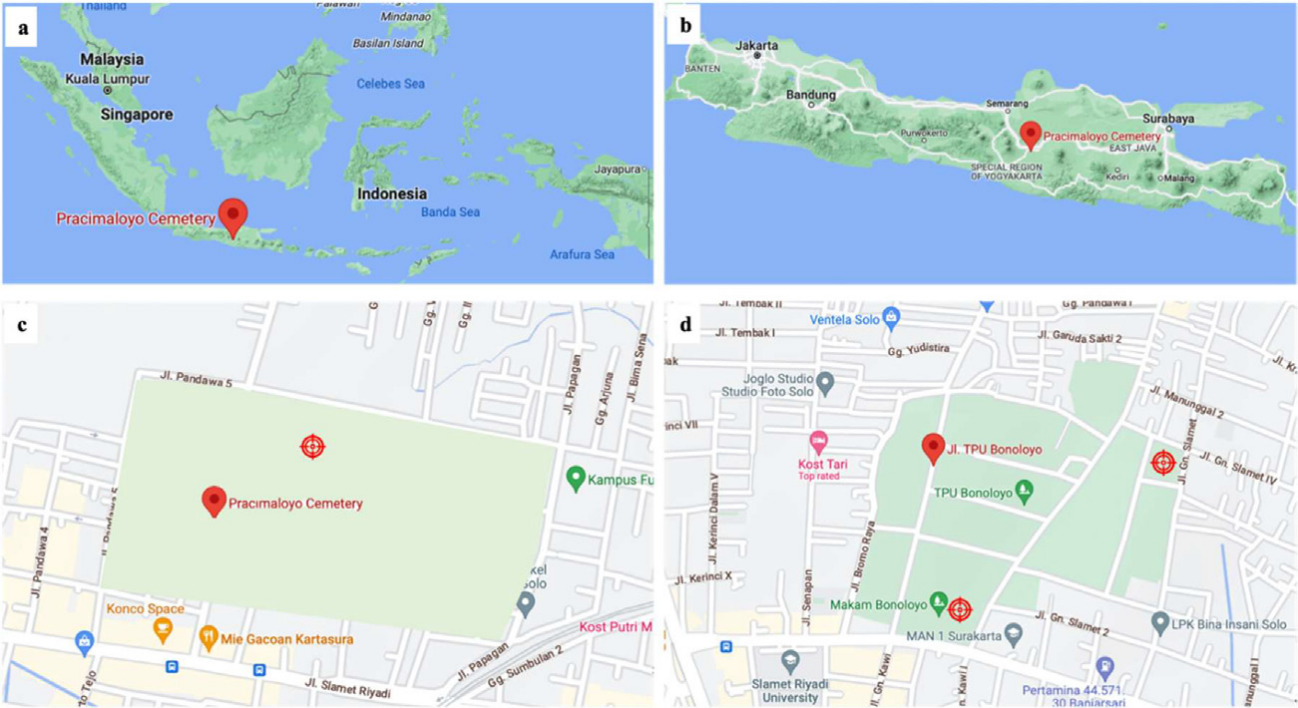
Maps of Pracimaloyo and Bonoloyo Cemeteries (green areas). (a) Map of Indonesia, (b) Java Island, the two cemeteries are in Central Java, (c) Pracimaloyo Cemetery, (d) Bonoloyo Cemetery. Red circle icons () indicate the sampling points. The maps were retrieved from google map.

Soil samples were excavated from two distinct cemetery sites located in Surakarta City, Central Java, Indonesia. Each cemetery area comprised three designated sampling points, each of which was sampled at two different depths. The sampling point was in between two personal graves with the distance of 15 cm from the personal grave. The sampling point was clear from the annual plantation and was colonized with the shrubs (Fig. 1). The geographical layout of these sampling points is visually depicted in Fig. 2. Subsequently, the collected soil samples underwent comprehensive chemical and physical analysis. The outcomes pertaining to the chemical composition and physical characteristics of the sampled soils are meticulously detailed in Table 1. Despite the rigorous analysis, discernible distinctions between the soils from different depths (surface and deep) were not readily apparent.

**Table 1:** The results of chemical analysis of collected cemetery soils.

Parameters	Unit	Areas and depths of sampling (cm)						Methods
		B18		B23		P12		
		20 cm	140 cm	20 cm	140 cm	20 cm	140 cm	
pH (KCl)		6.69	6.27	6.54	6.87	7.39	6.45	pH meter 1:5 IK. 5.4.c
Electrical conductivity	(µs/cm)	87	28	13	18	9	42	Conductometer 1: 5
C-organic	%	1.23	1.03	1.13	0.99	0.79	0.73	Walkly & Black IK 5.4.d
Total-N	%	0.08	0.07	0.04	0.05	0.06	0.03	Kjeldahl IK 5.4.e
Available K	ppm	106	91	129	124	147	171	Morgan-Wolf
P₂O₅	ppm	2	1	N/A	N/A	41	93	Olsen IK.5.4.h

**Fig. 3:**
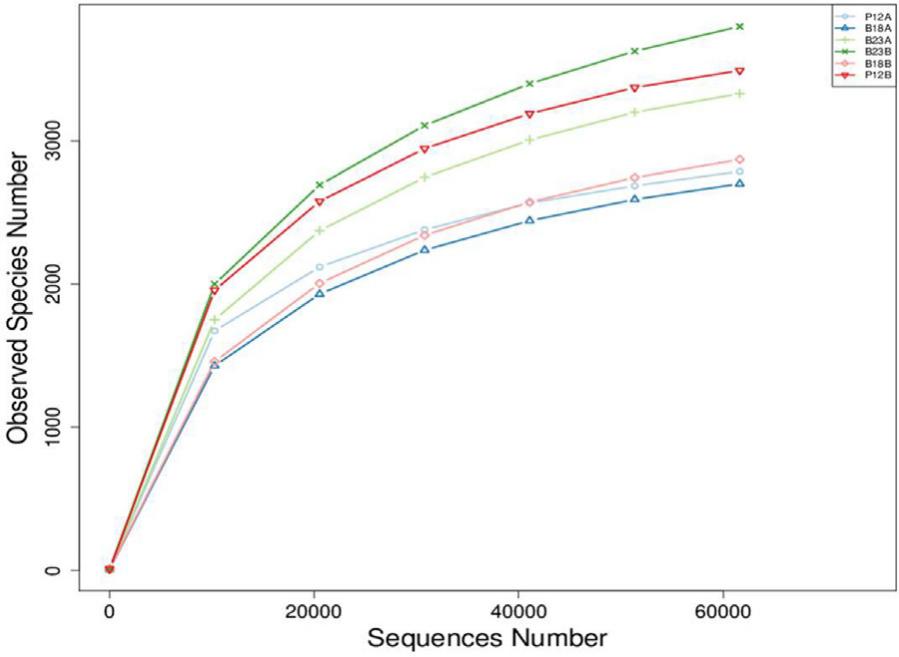
Number of Observed OTUs in the soil samples of two Cemetery areas in Surakarta City.

The data consists of the raw partial sequence of 16S rRNA of total bacteria in the Cemetery soils at 20 cm (A group) and 140 cm (B Group) depths. The statistical quality control showed that the average lengths of the samples ranged from 415 to 418 nucleotides (nt). The total raw reads from the sequencing ranges from 32,485,253 to 68,513,624 bases. The details of sequences quality can be seen in Table 2. Additionally, the rarefaction curves tended to approach the saturation plateau in all the six samples (Fig. 3). Rarefaction curves demonstrated that the abundance of operational taxonomic unit (OTU) was diverse among different samples.

**Table 2:** Statistical quality control of the raw data. P and B codes indicate the Pracimaloyo and Bonoloyo Cemetery grounds, respectively. While A and B letter correspondingly indicated the 20 cm-depth and 140 cm-depths.

Sample code	Raw PE(#)	Raw Tags(#)	Clean Tags(#)	Effective Tags(#)	Base(nt)	AvgLen (nt)	Q20	Q30	GC%	Effective %
P12A	176,256	126,635	120,477	77,857	32,485,253	417	96.55	89.91	58.08	44.17
P12B	170,959	147,164	142,209	95,022	39,564,449	416	97.70	92.89	56.83	55.58
B18A	157,426	125,678	118,409	97,677	40,850,657	418	97.73	92.88	54.90	62.05
B18B	188,382	182,059	179,962	164,563	68,513,624	416	98.30	94.56	57.28	87.36
B23A	183,052	160,517	155,828	105,118	43,643,302	415	97.69	92.89	56.64	57.43
B23B	181,809	160,031	155,707	119,412	49,598,251	415	97.64	92.73	56.79	65.68

**Fig. 4:**
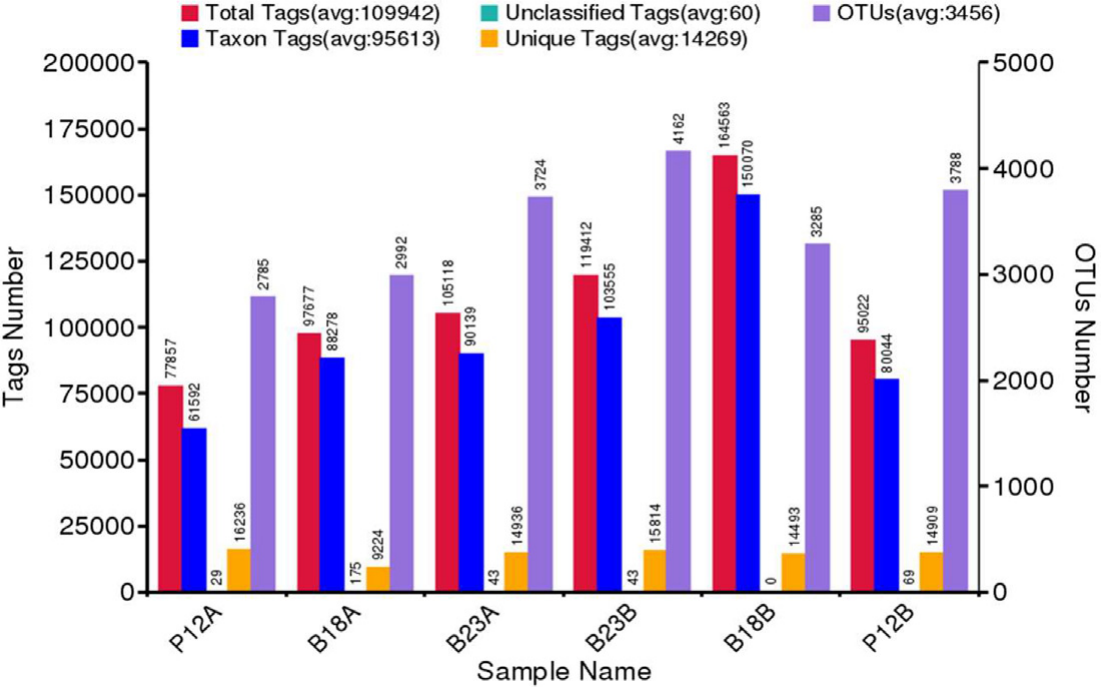
Summarization of the tags and OTUs number of each sample.

**Fig. 5:**
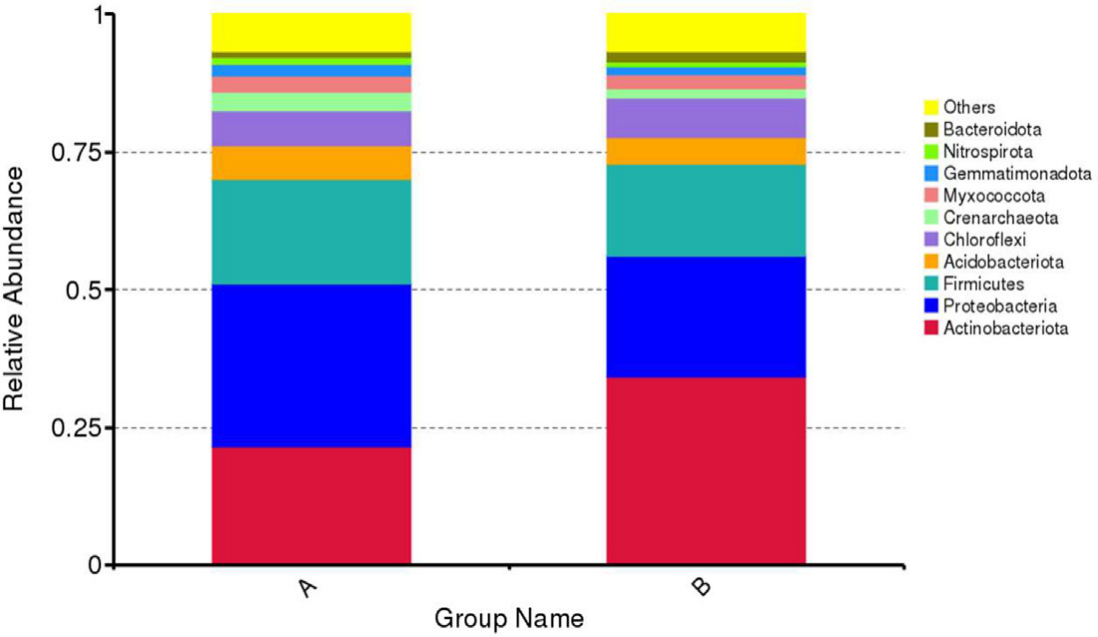
Phylum distribution of bacteria in the two cemetery grounds based on the depth. A = 20 cm; B = 140 cm.

**Fig. 6:**
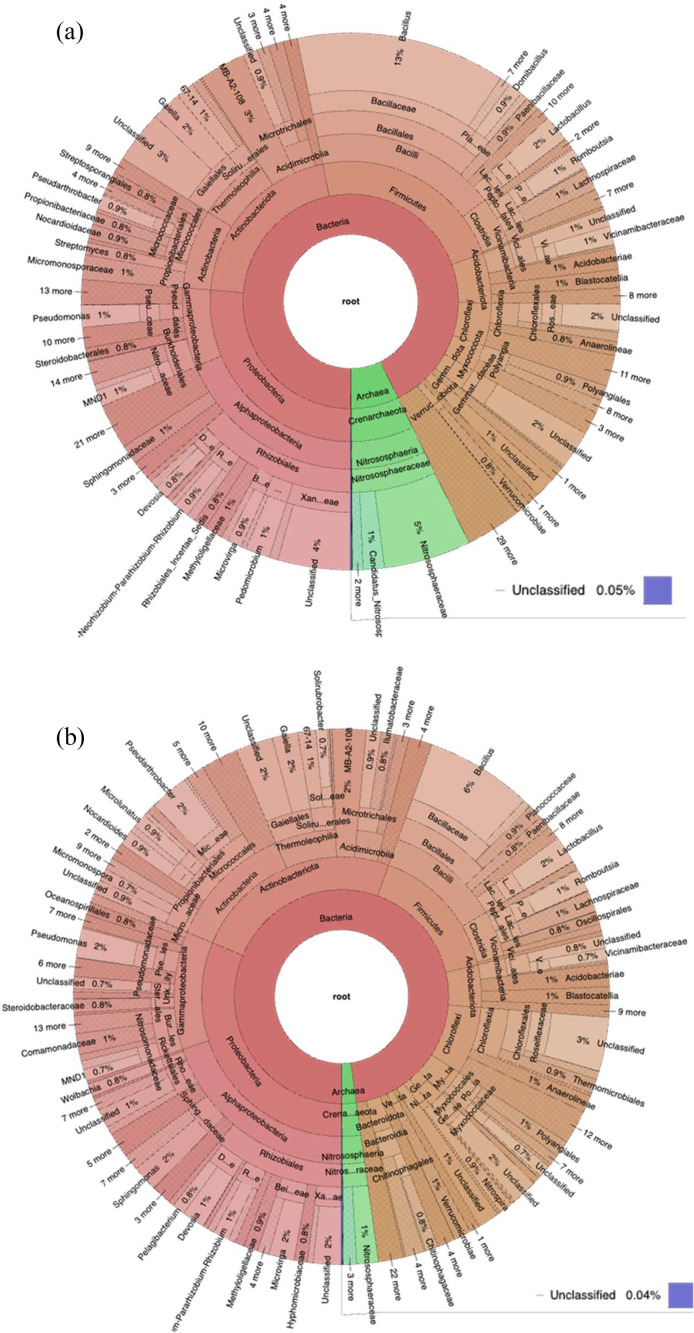
Krona plot showing the representative of bacterial distribution. (a) B23A sample indicated the bacterial distribution from soil sample in 20 cm depth, while (b) showed B23B bacterial distribution from soil sample in 140 cm depth.

The number of OTU and sample taxon of group B (140 cm) was greater than that of group A (20 cm) (Fig. 4 and Table 3). Each taxon level, from kingdom to species, was more numerous in group B at all sampling points, namely P12, B18, and B23 except at point B18 species taxon level (Table 3). Moreover, based on the depth of soil samples, Actinobacteria dominated with 34.2 % at a depth of 140 cm followed by Proteobacteria (21.9 %) and Firmicutes (16.6 %), while at a depth of 20 cm Proteobacteria were more dominant (29.5 %) than Actinobacteria (21.6 %) and Firmicutes (19.2 %) (Fig. 5). In addition, bacterial distributions in the soil samples are described in the Krona Plots at the level of bacterial genus (Fig. 6). A brief description showed that Phylum Actinobacteria were more dominant at the deep point of soil than the surface one. Meanwhile at the surface of soil were relatively dominated by Phylum Firmicutes than Actinobacteria.

**Table 3:** Number of taxa between samples.

Sample	Kingdom	Phylum	Class	Order	Family	Genus	Species
P12A	61592	60221	59109	56476	51313	40371	2513
P12B	80044	79315	78093	76537	71335	58172	10015
B18A	88278	87551	87052	85591	78148	70597	13900
B18B	150070	147909	146530	144821	129219	108450	12875
B23A	90139	89011	88031	86482	80967	67259	13034
B23B	103555	102197	101148	99350	93701	75493	18277

The total raw data has been archived in NCBI with project ID PRJNA997385. The sample NCBI sample IDs are SAMN36678868 and SAMN36678869 for 20 cm and 140 cm depths, respectively. Overall, the Sequence Read Archive (SRA) data can be accessed within this following link https://www.ncbi.nlm.nih.gov/sra/PRJNA997385.

## 3 Experimental Design, Materials and Methods

### 3.1 Sample collection and DNA extraction

Soil samples were collected from two cemetery grounds, Pracimaloyo (P) and Bonoloyo (B), in Surakarta City, Central Java, Indonesia. The samples were picked up from 20 cm-depth (A group) and 140 cm-depth (B group). Out of six total samples, two samples from P ground were collected from 20 cm-depth and 140 cm-depth then were labeled as P12A and P12B, respectively. Whereas four samples from B ground were collected in the depth of 20 cm-depth and 140 cm-depth and accordingly labeled as B18A, B23A, B18B, and B23B. The total genetic material of soil samples was extracted using the ZymoBIOMICS DNA Miniprep Kit according to the manufacturer's protocol.

### 3.2 Bacteria community analysis

Analysis of bacteria community was carried out using Illumina HiSeq 2500 PE250 (Novogen, Korea) based on the V3–V4 hypervariable regions of 16S rRNA and amplified using 341F/R806 primer sets [1,2]. The sequence of the primer sets was 341F 5′-CCTAYGGGRBGCASCAG-3′ and 806R 5′-GGACTACNNGGGTATCTAAT-3′. All PCR reactions were conducted with Phusion High-Fidelity PCR master mix (New England Biolabs). The DNA amplification through PCR involved an initial denaturation at 98 °C for 2 minutes, followed by 35 cycles of annealing starting at 65 °C and ending at 55 °C for 15 seconds, with extension at 68 °C for 30 seconds. The temperature for annealing was reduced by 1 °C per cycle until it reached 55 °C [1].

### 3.3 Quantification and qualification of polymerase chain reaction products

Same volumes of 1X loading buffer (contained SYBR green) were mixed with PCR products and run on 2 % agarose gel electrophoresis for detection. Samples with a bright main strip in the range 400–450 bp were chosen for further experiments.

### 3.4 Sequencing library preparation

Sequencing libraries were generated using the NEBNextⓇ UltraTM DNA Library Pre Kit for Illumina, following the manufacturer's recommendations and index codes were added. The library quality was assessed using a QubitⓇ 2.0 Fluorometer (Thermo Scientific) and an Agilent Bioanalyzer 2100 system. Finally, the library was sequenced on an Illumina platform and 250 bp paired-end reads were generated.

### 3.5 Data analysis

The triplicate samples from each depth consisting of 20 cm (A group) and 140 cm (B group) were combined. The data from each paired-end sequenced reading were combined using the FLASH software [3] which then produced raw tags data which were filtered based on the QIIME (v1.7.0) software [4] to obtain clean (high-quality) tags [5]. High quality tags were obtained by filtering the raw tags using the UCHIME algorithm and clustered into operational taxonomic units (OTU) using a cutoff percentage of bases with the quality score > 20 and error rate < 0.01 (Q20). The clean tags were compared to databases (Gold database) using the UCHIME algorithm [6] to detect chimera sequences which were then removed to obtain effective tags [7]. Sequenced data (effective tags) were then analyzed using the UPARSE software [8]. Sequences with a similarity ≥ 97 % were grouped into the same OTU. Afterward, each OTU was compared with the SILVA 132 database (https:// www.arb-silva.de/) to annotate species at each taxonomic rank (threshold: 0.8–1).

## Limitations

Not applicable.

## Ethics Statement

All authors have thoroughly adhered to the ethical guidelines for publication in Data in Brief. We affirmed that the present study does not involve human subjects, animal experiments, or the collection of data from social media platforms.

## CRediT authorship contribution statement

**Triastuti Rahayu:** Conceptualization, Supervision, Resources, Funding acquisition, Formal analysis, Writing – original draft, Project administration, Methodology, Data curation. **Erma Musbita Tyastuti:** Project administration, Methodology, Data curation. **Ambarwati Ambarwati:** Methodology. **Lina Agustina:** Methodology, Data curation. **Noor Alis Setiyadi:** Methodology, Data curation. **Nazia Jamil:** Supervision, Writing – review & editing. **Yasir Sidiq:** Methodology, Investigation, Formal analysis, Writing – review & editing.

## Data Availability

Cemetery soil Raw sequence reads (Original data) (NCBI SRA).
